# The Influence of Different Parameters for the Removal of Pb and Zn Ions on Unmodified Waste Eggshells

**DOI:** 10.3390/ma18122794

**Published:** 2025-06-13

**Authors:** Elena Petronela Bran, Oana-Irina Patriciu, Luminița Grosu, Irina-Claudia Alexa, Brîndușa Bălănucă, Adrian-Ionuț Nicoară, Adriana-Luminița Fînaru

**Affiliations:** 1 Doctoral School, “Vasile Alecsandri” University of Bacău, 157, Calea Mărăşeşti, 600115 Bacău, Romania; petronelabran@yahoo.com; 2 Department of Chemical and Food Engineering, Faculty of Engineering, “Vasile Alecsandri” University of Bacău, 157, Calea Mărăşeşti, 600115 Bacău, Romania; oana.patriciu@ub.ro (O.-I.P.); lumig@ub.ro (L.G.); 3 Department of Organic Chemistry “C. Nenitescu”, Faculty of Chemical Engineering and Biotechnologies, National University of Science and Technology Politehnica Bucharest, 1-7, Gheorghe Polizu Street, 011061 Bucharest, Romania; brindusa.balanuca@upb.ro; 4 Department of Science and Engineering of Oxide Materials and Nanomaterials, Faculty of Chemical Engineering and Biotechnologies, National University of Science and Technology Politehnica Bucharest, 1-7, Gheorghe Polizu Street, 011061 Bucharest, Romania; adrian.nicoara@upb.ro; 5 National Research Center for Micro and Nanomaterials, National University of Science and Technology Politehnica Bucharest, 060042 Bucharest, Romania

**Keywords:** food waste, heavy metal, biosorption, agitation-activation system

## Abstract

The need to use environmentally friendly and cost-effective methods to remove heavy metals from wastewater is a permanent concern worldwide. Eggshells have been indicated as a worthy biosorbent for the adsorption of heavy metals due to their bioavailability and composition. In the present study, the absorption capacity of untreated chicken (CEs) and quail (QEs) eggshells for the removal of Pb and Zn ions from aqueous solutions was evaluated at room temperature and 40 °C, using four types of agitation systems: classical and orbital agitation and ultrasonic and microwave-assisted activation. The monitoring of aqueous solutions was performed by electrochemical and spectro-analytical (AAS) procedures before and after the adsorption process. FTIR and RAMAN spectroscopy, SEM-EDAX microanalysis, and X-ray diffraction were used to investigate the characteristics of eggshell samples post-exposure to Pb^2+^ or Zn^2+^. For any type of agitation and temperature, the CEs were able to induce more than 65% removal efficiency for lead and over 80% in the case of zinc. Concerning the Zn removal efficiency of QEs, notable results were recorded when microwaves were applied (>90%) and at 40 °C for orbital shaking and ultrasound (>80%). The results of the present study may offer new and valuable information for the optimal removal of Pb^2+^ and Zn^2+^ using eggshells, thus contributing to the sustainable management of waste through the recycling of this type of biomaterial.

## 1. Introduction

Urbanization and industrialization activities are constantly growing, playing an essential role in the economic flourishing of any country. However, these activities unfortunately lead to environmental pollution.

On the other hand, all over the world, the food processing industry generates voluminous by-products and waste materials. Consequently, there is a constant concern for the conversion of waste, and its valorization can help to offset growing environmental problems and facilitate the sustainable use of available natural resources [1].

Eggshell is an example of specific waste in the food sector; the processing industry is facing the challenge of proper management of eggshells, both from economic and environmental points of view [2].

According to the official website of the European Union, Romania is the seventh country in terms of both annual egg production and consumption [3].

In Bacău County (Romania), the food industry is very well represented; Agricola International Bacău is a nationally and internationally known group that offers, among other things, chicken eggs for consumption (trades, restaurant, catering activities, etc.) and egg powder (for patisserie, confectionary, other food industry, etc.) [4]. At the same time, lately, many quail egg farms have been developed in the region because consumer demand for this product has increased [5].

Taking into account that one kilogram of egg powder is made from 90 chicken eggs [4], and that the household consumption of chicken and quail eggs is constantly increasing, finding viable solutions for the valorization of eggshells is particularly important.

Worldwide, the development of an appropriate strategy for the valorization of eggshell biowaste has been considered a challenge for the food industry in terms of environmental protection, not only due to the large quantities generated but also to its high potential for microbial proliferation [6,7].

The idea of revaluing eggshells is not new and, over the years, many studies have proposed different alternatives for their valorization: the production of food additives [2,6], material for supplements, cosmetics and pharmaceutics [6,8,9], soil fertilizer [10,11,12], biomaterial composites, etc. [2].

One of the well-known applications of eggshells studied in the literature is their use as low-cost and environmentally friendly biosorbent materials for the removal of heavy metals from contaminated waters and soils [13,14,15].

Currently, environmental pollution has become a major problem worldwide. Heavy metals are continuously released into the biosphere from natural causes (e.g., volcanic eruptions) and from the development of many industries (chemical, metallurgical, mining, food, agricultural, etc.), leading to the appearance of many toxic compounds in the air, soil, and water, with extremely damaging effects on both the environment and human health. Pb, Zn, Cd, and Ni are among the dangerous heavy metals that can be found in various water sources [16,17,18].

Numerous methods have been applied for the removal of heavy metals from water and wastewater, involving adsorption, chemical precipitation, electro-dialysis, ion exchange, microfiltration, reverse osmosis, ultrafiltration, etc. [19,20,21].

Contrary to some methods that have sometimes proven unsuitable to be applied in industries and involve high costs, the bioadsorption process is based on the ability of metals to bind to the surface of various biological materials used as biosorbents and constitutes a reliable and economical method. Thus, scientists have increasingly focused their attention on it in order to eliminate heavy metals from water. The bioadsorption of heavy metals from wastewater has been studied over time using various types of waste, such as peanut shells, banana peels, orange peels, sunflower seed shells, oyster shells, coffee grounds, etc. [22,23,24,25,26,27,28]. The use of agri-food waste as a biosorbent is cost-effective because, in addition to the fact that it valorizes the waste, it does not require extensive processing technology and it has a good adsorption capacity.

Many studies have used eggshells to remove various pollutants from water or wastewater, including heavy metals such as cadmium, copper, iron, lead, and zinc [20,29,30]. Due to its composition (high proportion of calcium carbonate of over 97%), as well as its bioavailability, eggshells have proven to be a good biosorbent responsible for removing metals [31]. It is well known that calcium carbonate interacts strongly with some divalent metal ions (Me^2+^) and the removal of metal ions in solution can be carried out by adsorption [32,33]. The effect of various parameters affecting the adsorption of heavy metals using eggshells as biosorbents including ratio sorbent/metal solution, initial metal concentration in aqueous solution, sorbent particle size, pH, temperature, contact time, etc. has been already described [2,34,35,36].

According to the Regional Agency for North-East Environmental Protection (Romania), heavy metal pollution has been reported in Bacău County due to industrial polluters, the intensification of road traffic, etc. Lead (Pb) and zinc (Zn) are pollutants coming from various industrial sources that reach surface waters and represent a real danger to human health but also to flora and fauna. As a permanent interest of our research team, in a previous study, we investigated the accumulation of heavy metals in different species of fish coming from an area influenced by the industrial platform of the city of Bacău situated at the confluence of Bistriţa River and Siret River [37].

It is generally recognized that the agitation influences the mass transfer from the solid to the liquid. An important parameter in the adsorption process, the mixing rate allows for increasing the removal efficiency by intensification of the contact between the adsorbent and ions [38]. As far as we know, a small number of studies have focused on the effect that different stirring techniques (e.g., agitation or shaking) can have on the adsorption process using eggshells as bioadsorbents [36,39]. However, no study has been reported comparing multiple types of agitation/activation in order to remove heavy metals from aqueous systems using eggshells.

The objectives and novelty points of the present study are as follows:

(i) Explore the influence of experimental conditions on the adsorption efficiency of chicken and quail eggshells using four types of agitation/activation systems for the adsorbent solution samples: classical and orbital agitation, ultrasonic, and microwave activation.
(ii) Compare the adsorption capacity of chicken and quail eggshells for the removal of Pb^2+^ and Zn^2+^ from aqueous solutions, without any preliminary modification on the eggshells such as calcination or structural changes.
(iii) Analyze the morpho-structural characteristics of eggshell powder samples post-exposure to heavy metal ions using FTIR and RAMAN spectroscopy, SEM-EDAX microanalysis, and X-ray diffraction (XRD).
(iv) Improve the bioadsorption process of heavy metal ions, by applying environmentally friendly activation methods, aiming to reduce time and energy consumption, while maintaining high adsorption capacities, which was rarely reported.

## 2. Materials and Methods

All chemicals used were analytical reagent grade and purchased from Sigma Aldrich Chemical Company (Darmstadt, Germany).

The stock solutions containing the Pb^2+^ and Zn^2+^ ions (1000 mg·L^−1^) were prepared from Pb(NO_3_)_2_ and Zn(NO_3_)_2_·6H_2_O, respectively. In order to reach the required amounts of ion metals, the dilution with bidistilled water was performed.

### 2.1. Preparation of Unmodified Eggshell-Based Biosorbent

The chicken eggshells used in this study (noted CE_S_) were purchased from Agricola International Bacău, Romania [4], and the quail eggshells (noted QE_S_) from a microfarm from Petru-Vodă, Neamț, Romania [5].

**Figure 1:**
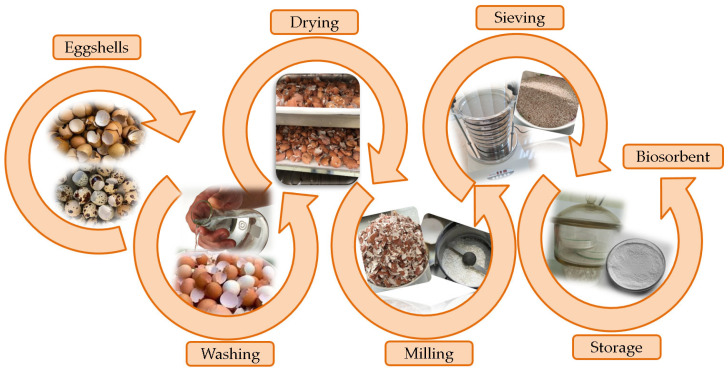
Schematic diagram of preparation of unmodified eggshell-based biosorbent.

The preparation of eggshells for the experiments was carried out according to a well-established protocol within the laboratory, schematically presented in Figure 1.

**Figure 2:**
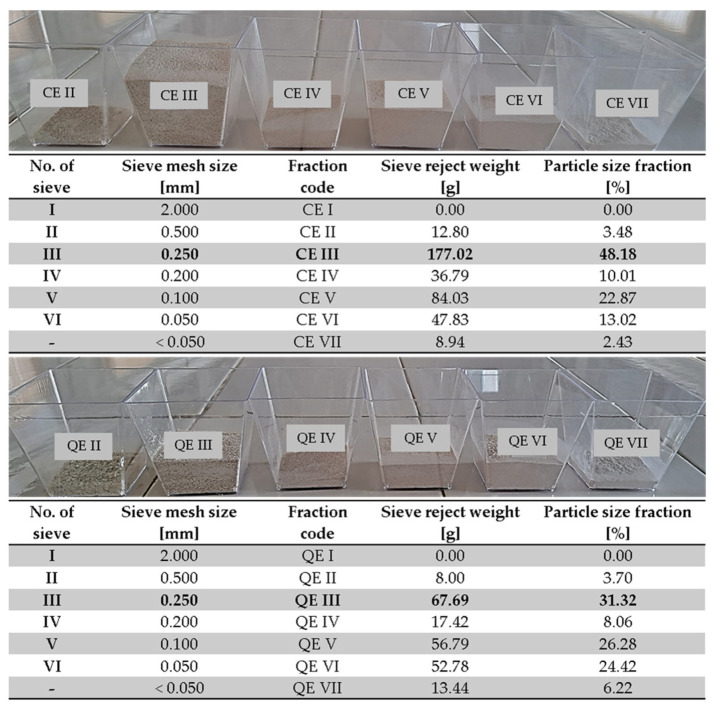
Fractions of eggshell powder after the sieving process.

The eggshells were washed with tap water to eliminate impurities, rinsed with distilled water, and then dried in an air circulation oven (Memmert Universal, model UFE 500, Memmert GmbH+Co.KG, Schwabach, Germany) at 100 °C for 30 min. The dried eggshells were then milled for 3 min using an electric grinder (Heinner, model HCG-150SS, 150 W, Heinner, Bucarest, Romania). Subsequently, the powder obtained was sieved using a digital electromagnetic sieve shaker (Filtra Vibración, model IRIS FTS-0200, Barcelona, Spain) [30,38]. The resulting eggshell powders were classified into seven groups depending on particle size (Figure 2).

From the seven fractions obtained, the major fractions (fraction III—sieve mesh size, 0.250 mm) were chosen as biosorbents, placed in Petri dishes, and stored in a desiccator until their use in the biosorption process.

### 2.2. Description of Biosorption Process

In order to establish the optimal parameters (initial concentration of the metal ion, biosorbent amount, solution pH, contact time) for the following bioadsorption experiments, and based on data described in the literature [40], a preliminary investigation was conducted using chicken eggshells as biosorbent, lead ions solution as adsorbate and classical agitation with a magnetic stirrer (data shown in Supporting Information—Figures S1–S3).

As a result of this preliminary study, the following values were adopted for the investigated parameters: metal ion concentration = 5 mg·L^−1^, adsorbent concentration = 2 g·L^−1^, pH = 5, contact time = 60 min. The pH value was corrected using 0.1 N HCl to reach the optimal pH value. In addition, the pH_pzc_ = 4.63 (Figure S4 from Supporting Information) and optimal pH value resulting from our preliminary study (pH = 5) are in agreement with the results reported by Praipipat et al. for chicken eggshell powder [39], respectively, at pH_pzc_ = 4.47 and the highest lead removal efficiency at pH = 5.

In the present study, the adsorption experiments were carried out in 250 mL Erlenmeyer flasks containing 200 mL of 5 mg·L^−1^ metal ions (Pb^2+^ or Zn^2+^) and 0.4 g of previously prepared powder eggshells. Batches were performed using chicken and quail eggshell powder separately, as well as with a mixture of the two in equal proportions.

Four types of agitation/activation techniques were tested aiming to increase the efficiency of the adsorption process and reduce the impact on the environment. Thus, the experiments were performed at room temperature (r.t.) and 40 °C, respectively, under all the conditions presented in Table 1.

After the adsorption process, the samples were filtered through Whatman filter paper no. 42 (Marlborough, MA, USA). Both the filtrate and the solid residue were subjected to further analysis.

### 2.3. Characterization of Aqueous Solutions Before and After the Adsorption Process

The aqueous solutions of the analyzed ions were characterized by electrochemical methods before and after the adsorption process.

For each solution sample, electrochemical parameters were determined: pH, EC—conductivity, SAL—salinity, and TDS—total dissolved solids. The determinations were performed in triplicate using Thermo Scientific™ Orion™ Versa Star Pro™ Multiparameter Benchtop Meter (Thermo Fisher Scientific, Waltham, MA, USA) provided with ROSS Ultra pH/ATC electrode and DuraProbe conductivity cell 013005MD.

The filtrates were analyzed for residual concentration of heavy metals using atomic absorption spectrophotometry (AAS) according to ISO 8288:1986 [41] in the water quality accredited laboratory (ISO 17025/2018) [42] from the Siret River Basin Water Administration (ABAS, Bacău, Romania). ABAS’s activity complies with the quality management standard ISO 9001:2015 [43].

The efficiency of adsorption can be measured by calculating the percentage removal of metal ions. The removal efficiency R was calculated with the given initial (C_0_) and final (C_e_) concentration of ions in solution (mg·L^−1^) following Equation (1).

R (%) = (C_0_ − Ce)/ C_0_ × 100, (1)

### 2.4. Characterization of Eggshell Powders Before and After the Biosorption Process

Fourier-transform infrared spectroscopy (FTIR), scanning electron microscopy (SEM), and energy-dispersive X-ray analysis (EDS) were used to investigate the modifications in functional groups and morphology of chicken and quail eggshell samples post-exposure to Pb^2+^ or Zn^2+^.

Fourier Transform Infrared Spectroscopy spectra were obtained using a Nicolet iS50N spectrometer (Thermo Fisher, Waltham, MA, USA) with attenuated total reflection (ATR). The scanning was performed between 4000 and 400 cm^−1^ at a resolution of 4 cm^−1^. Spectral recording was performed by connecting the spectrometer to a data acquisition and processing unit through the OMNIC work program.

The crystalline phase composition was assessed by X-ray diffraction analysis. Analysis was performed at room temperature using Panalytical Empyrean (Malvern Panalytical, Almelo, The Netherlands) equipment with radiation Cu K a = 0.154 nm, the scanning being performed between 2θ = 5–80°, with a scan step of 0.02° and time per step of 255 s and 45 kV and 40 mA. The average crystallite size (D) was determined using Scherrer’s Equation (2).

(2) $$D=\frac{k\cdot \lambda }{\beta \cdot \cos\theta }$$

where β is full width at half maximum (FWHM), θ is Bragg’s angle, λ is the wavelength of the X-ray, and D is the crystallite size.

The XRD data were analyzed using HighScorePlus 3.0e software, with a whole pattern profile fitting (WPPF) module, connected to the ICDD PDF5+ database.

RAMAN spectra were recorded using a LabRAM HR Evolution Confocal Microscope (Horiba, France) with a 514 nm argon ion laser. Data were collected and analyzed for the non-polarized radiation scattered at 90°, with an acquisition time of 5s during 10 runs recorded for each sample. All raw data obtained from structural investigations were plotted and interpreted using OriginPro 9.0 software (OriginLab, Northampton, MA, USA).

The microstructure of the studied materials was assessed using a Quanta Inspect F50 high-resolution electronic scanning microscope (1.2 nm resolution-Thermo Fisher, former FEI, Eindhoven, The Netherlands) with an energy-dispersive spectrometer (SEM-EDS). All the images were recorded using the back-scattered electron detector (BSE) with the accelerating voltage set at 30 kV.

The samples were affixed via carbon tape to the SEM sample holders and vacuum-coated for 40 s with gold. The elemental composition of materials before and after contact with aqueous solutions of the ions under study (Pb and Zn) was achieved at 30 kV and spot 5 using the energy-dispersive X-ray detector (EDS) produced by EDAX (Mahwah, NJ, USA).

### 2.5. Statistical Analysis

For establishing the relationship between different recorded parameters, Pearson correlation analysis was completed using Microsoft EXCEL 2010 Statistical Tool Package.

The graphical representations were generated using Origin 2024 10.1.0.170 Academic software.

Principal component analysis (PCA) and hierarchical cluster analysis (HCA) were performed using R-Commander (version 4.4.3) and FactoMineR plugins for multivariate analysis [44] to highlight the similarities or differences between the samples after the bioadsorption process.

## 3. Results and Discussion

### 3.1. Electrochemical Characterization of Aqueous Solutions Before and After the Adsorption Process and Removal Efficiency R

The initial solutions of heavy metal ions used in the present study were analyzed by electrochemical methods. The results of these determinations are presented in Table 2.

**Figure 3:**
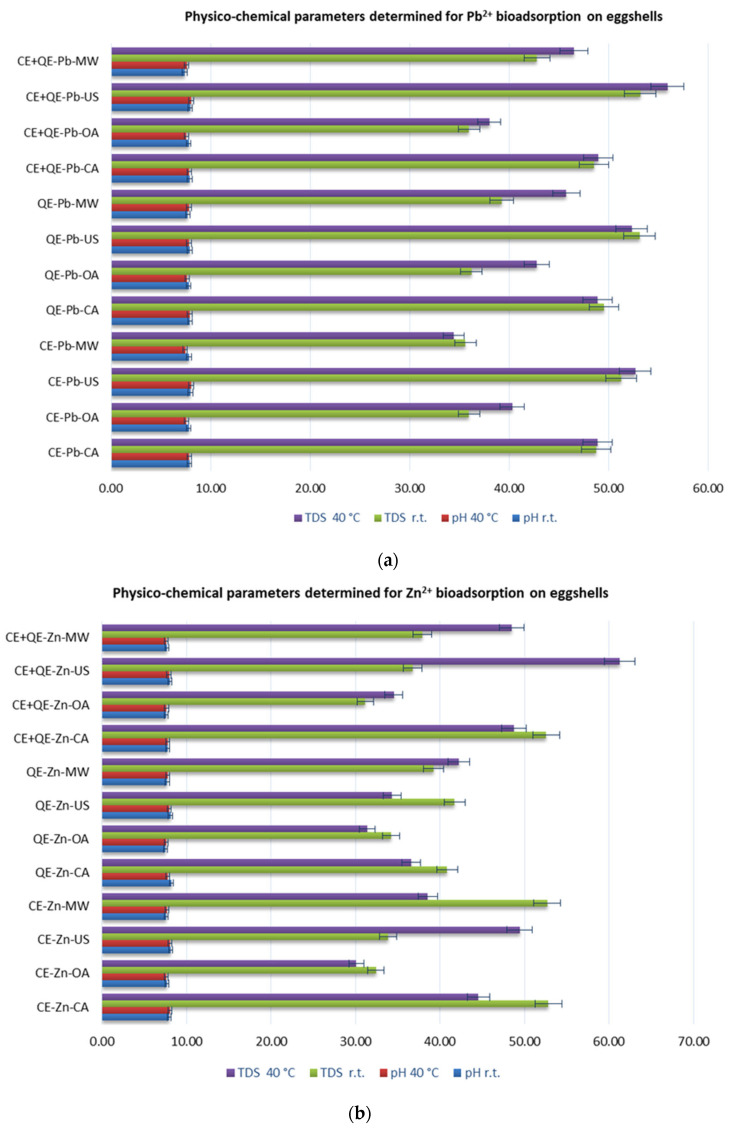
Physico-chemical parameters of aqueous solution for Pb^2+^ (**a**) and for Zn^2+^ (**b**) after adsorption.

Physico-chemical parameters of aqueous solutions after bioadsorption, including pH, electrical conductivity (EC), total dissolved solids (TDSs), and salinity (SAL) were monitored. Figure 3 (Tables S1 and S2 of Supporting Information) depicts the recorded parameters for Pb^2+^ and Zn^2+^, respectively.

Comparing the values obtained for Pb^2+^ and Zn^2+^ solutions after contact with the eggshell powders with those before the adsorption process, a change can be observed, indicating that there were interactions during the contact between the eggshells and the solution containing heavy metal ions.

Thus, it can be seen (Figure 3, Tables S1 and S2 of Supporting Information) that concerning the pH, the values have shifted from the acid zone (5.52–5.56) to the neutral to weakly basic zone (7.41–8.21), being comparable regardless of activation method or temperature, both for zinc and lead. Among all samples resulting from the Pb^2+^ adsorption process, the highest pH value (8.05) was recorded on chicken eggshell powder under ultrasound-assisted activation at 40 °C. The resulting solution in the case of the zinc ion adsorption process using quail shell powder, under classical stirring conditions (at r.t.), had a pH value of 8.21, the highest of all recorded values.

Changes in pH can be explained by the fact that in an acidic environment, some of the calcium carbonate present in the eggshell dissolves, releasing calcium and bicarbonate ions, which lead to more neutral to slightly alkaline values [45].

The values obtained for EC, TDS, and SAL after the bioadsorption process are much higher than the initial ones.

Analyzing the overall results of electrochemical parameters, it is noted that in the case of Pb solutions after the biosorption process using all types of eggshells (Figure 3a), the highest values for EC (over 100 µS·cm^−1^) and TDS (over 50 ppm) are registered when activation occurs with ultrasound, both at ambient temperature and at 40 °C.

**Figure 4:**
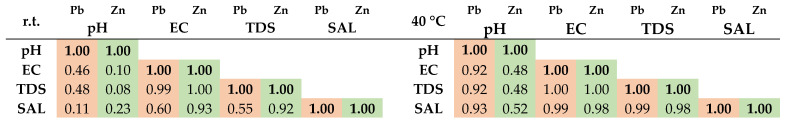
Pearson’s correlation for the physico-chemical parameters of aqueous solutions of Pb and Zn ions after adsorption.

The Pearson’s correlation coefficients calculated for the physico-chemical parameters of aqueous solutions of Pb and Zn are presented in Figure 4.

The results revealed very strong correlations (0.92–1.00) between EC and TDS, TDS and SAL, and EC and SAL for almost all samples whether it is about samples containing Pb or Zn, both at r.t. and at 40 °C. Only in the case of an aqueous solution of Pb at r.t., a moderate correlation between EC and SAL and TDS and SAL was perceived (0.60, respectively, 0.55). Very strong correlations (0.92–0.93) were also observed between pH and EC, TDS, and SAL, respectively, in the case of Pb solutions at 40 °C.

**Figure 5:**
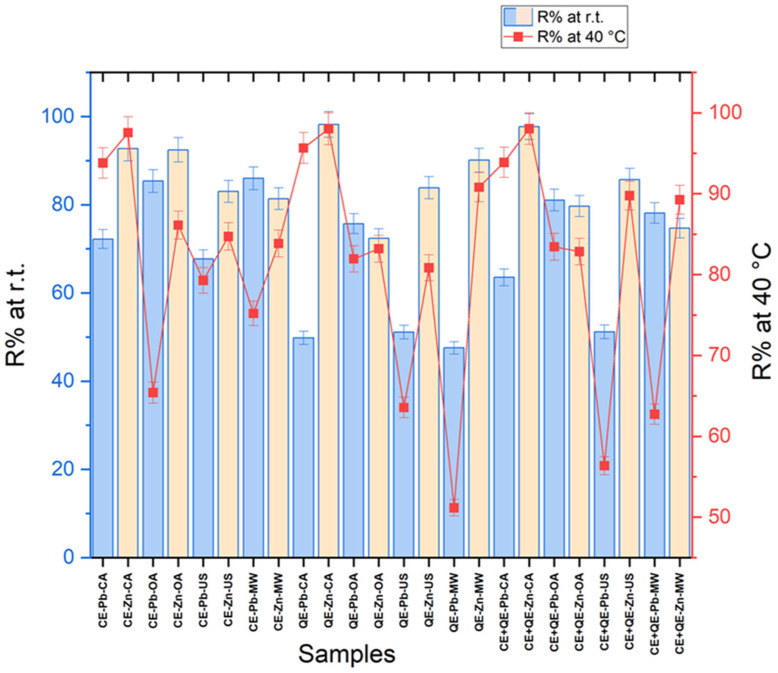
Eggshells removal efficiency (R) from the aqueous solution of Pb^2+^ and Zn^2+^, respectively.

The results of the removal efficiency (R, %) of Pb^2+^ and, Zn^2+^, respectively, from the aqueous solution by chicken eggshells, quail eggshells, and their mixture in equal proportions, for all types of agitation/activation, are graphically represented in Figure 5 (Table S3 in Supporting Information).

Analyzing the R values, it can be observed that the chicken eggshells were able to have more than 65% removal efficiency, whether it is the Pb or Zn ions, for any type of agitation and temperature. However, in the case of zinc, the efficiency has higher values (over 80%) than in the case of lead.

A percentage removal capacity of 56% was reported in the case of Pb ion adsorption on untreated chicken eggshells after 100 min of shaking [46].

Ahmad et al. described that the eggshells removal capability for Pb^2+^ after 24 h (conditions: thermal shaker at 25 °C and 120 rpm) ranged between 78.9 and 99.6% [40]. Compared to other biowaste materials with similar chemical composition (high CaCO_3_ content), chicken eggshells proved to possess a greater capacity to immobilize the Pb ion than, for example, oyster shell powder [28] or coral wastes [40].

Better results were observed in the case of classical agitation at 40 °C—97.6% for Zn^2+^ and 93.8% for Pb^2+^.

At room temperature, in the case of the Zn ion, very good results of over 92% were obtained in the case of both classical agitation and orbital shaking, respectively. These results are similar to those described by Hanifah et al. [47], which showed that calcined chicken and duck eggshells adsorb Zn^2+^ with a percentage removal of 97.6% also using a magnetic stirrer at room temperature for 60 min. In the study conducted by Badrealam et al., orbital shaking was used in the adsorption process of zinc on chicken eggshells, showing results of over 92% (60 min at 150 rpm) [30].

The use of ultrasound and microwaves in the adsorption process using chicken eggshells as biosorbents leads to good results in terms of adsorption capacity.

Concerning the removal efficiency of quail eggshells, the highest values (over 98%) were obtained using classical stirring for the Zn^2+^ adsorption, regardless of the temperature used. Good results (over 80%) were recorded at 40 °C when orbital shaking and ultrasound were used. Also, in the case of Zn, high values of R (over 90%) can be noted when microwaves are applied as an activation technique.

Mashangwa et al. studied the ability of chicken eggshells to remove lead and zinc ions from solutions, without any agitation technique. Their results showed that the equilibrium point for adsorption in the case of Pb^2+^ was attained after 120 min (98.3%) and for Zn^2+^ after 270 min (81.2%) of contact time [48]. The results obtained in our study for Zn ion adsorption show that introducing an agitation technique can lead to similar results (R over 80%) by reducing the time from 270 min to 60 min (CA), 30 min (OA and US), respectively, and 3 min (MW).

Also, in our experiments on Pb adsorption by classical stirring at 40 °C, good results of over 93% were obtained after only 60 min, equally in the case of using chicken, quail eggshells, and a mixture of the two types, respectively.

In the case of Pb adsorption, it can be observed that quail eggshells are less efficient than chicken eggshells, especially at room temperature (49.8–75.7%). The removal efficiency increased a lot from 49.8 to 95.6% upon classical stirring from room temperature to a temperature of 40 °C.

Unexpectedly small values (47.5 and 51.2%) were obtained when using microwave activation for the bioadsorption of Pb^2+^ on quail eggshells.

The mixture in equal proportions of chicken eggshells and quail eggshells led to good Zn adsorption in all experimental conditions with R values ranging between 74.7 and 98%. In the case of the Pb ion, the adsorption process on the eggshell mixture proceeded with an efficiency of 93.9% using classical stirring at 40 °C, while results of over 80% were obtained in the case of orbital activation regardless of temperature.

The results obtained for all types of biosorbents used indicate that, in the case of classical agitation, temperature favors the adsorption process of both Pb ions and Zn ions, which is in accordance with other studies [20,45,49].

At room temperature, improvements in the removal efficiency of the Pb ion from 72.2 to 85.4% (for CEs), from 49.8 to 75.7% (for QEs), and from 63.5 to 81.1% (for mixture CEs+QEs) were observed in the case of orbital agitation compared to classical stirring. It seems that the orbital shaking improves the diffusion of Pb ions toward the surface of the eggshells regardless of their type. However, in the case of the zinc ion, the same trend was not observed.

Ultrasonic activation of solutions subjected to the adsorption process allowed to achieve good results, especially in the case of Zn ion, for all biosorbent types (R = 80.8–89.8%).

While using the microwave-assisted activation for adsorption tests, the performances for removal efficiency are maintained at relatively good levels especially for the Zn ion (74.7–90.8%), regardless of the biosorbent (CEs, QEs, and mixture) and the power (170 and 340 W) used. In the case of Pb^2+^, the best results were recorded using chicken eggshells in the bioadsorption process, followed by the mixture, respectively, and by quail eggshells, for which values of only 47.5 and 51.2% were obtained.

It is well known that the cavitation effect, in the case of ultrasound, and heating energy in the case of microwaves, leads to an increase in agitation at the molecular level, drastically improving the mass transfer between the bioadsorbent and the metal ion solution [50,51,52].

**Figure 6:**
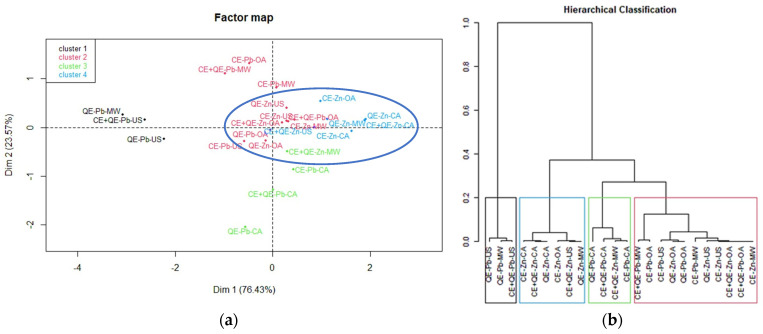
(**a**) Score plot determined by principal component 1 versus principal component 2 for the discrimination of all samples by removal efficiency R. (**b**) Hierarchical cluster analysis dendrogram for the discrimination of all samples by removal efficiency R.

Analyzing the dataset for the adsorption of studied heavy metals (24 samples, 4 variables: eggshell type, agitation mode, temperature, type of metal ions), similarities and differences observed between the samples are represented in Figure 6 (Tables S4 and S5 from Supporting Information).

The factor map resulting from the application of PCA (Figure 6a) offers a useful interpretation of the relationships and correlations among samples. Also, the hierarchical cluster analysis (Figure 6b) allows the formation of groups based on their similarities. From the score plot, it can be observed that the samples related to Zn adsorption are more confined than the Pb samples (blue circle in Figure 6a). The 24 samples were split into four clusters and their hierarchical classification is in excellent agreement with the PCA results.

The first cluster (in black) gathered three samples (QE-Pb-US, QE-Pb-MW, and CE+QE-Pb-US), for which the R values were the lowest (47.5–63.6%). These samples are positioned far from the others on the PCA score plot. Therefore, it can be stated that quail eggshells have a low efficiency in removing lead ions using ultrasound or microwaves as an activation system. Cluster 2 (in red) includes samples that led to removal efficiencies of heavy metal ions ranging from 62.7 to 86%. It can be seen on the PCA score plot that most of these samples are close to those from cluster 4 comprising the best results of removal efficiency. Cluster 3 (in green) contains samples in which the removal efficiency had a significant increase going from values between 49.8 and 77.2% at r.t. to values between 89.3 and 95.6% for 40 °C, especially in the case of lead adsorption using classical agitation. The samples regarding Zn ions removal efficiency with great values between 85.7 and 98.2% belong to cluster 4 highlighted in blue, showing that very good and similar results can be obtained using orbital agitation, ultrasound, or microwave activation compared to those obtained by classical agitation, regardless of temperature conditions.

### 3.2. Characterization of Eggshell Powders Before and After the Biosorption Process

**Figure 7:**
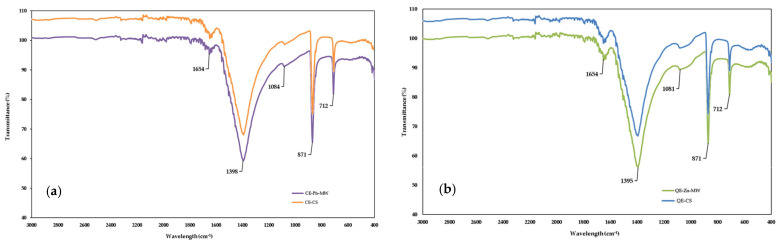
FTIR spectra of chicken (**a**) and quail (**b**) eggshell powder.

The change in functional groups of eggshell powder before and after Pb^2+^ removal was examined using FTIR spectroscopy. Figure 7a shows the overlapping FTIR spectrum of chicken eggshells before (control sample: CE-CS) and after the adsorption of Pb^2+^ under microwave activation (CE-Pb-MW), while Figure 7b shows those of quail eggshells before (control sample: QE-CS) and after the adsorption of Zn^2+^ under microwave activation (QE-Zn-MW), respectively.

The spectra of eggshell powders were in accordance with the previously reported results [20,53,54,55].

The eggshell powder samples presented similar spectra, regardless of their type and the ion metal. The most significant peaks were found at 1395 and 1398 cm^−1^, strongly associated with the presence of carbonate minerals within the eggshell matrix [56]. Furthermore, the two well-defined bands at approximately 871 and 712 cm^−1^ are, respectively, attributed to the asymmetric stretching and out-of-plane and in-plane deformation modes of the carbonate group in CaCO_3_ [57].

**Figure 8:**
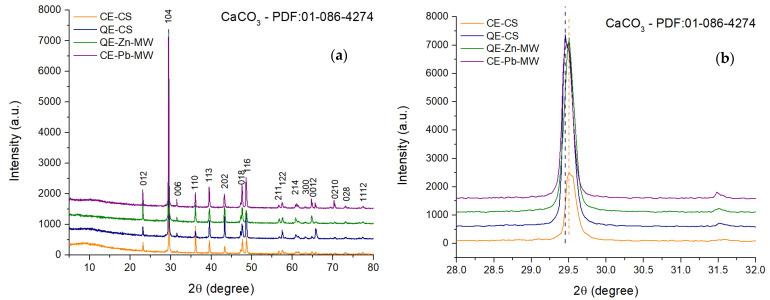
XRD pattern of chicken and quail eggshell powder (**a**) and CaCO_3_ (104) characteristic peak shift represented by dotted lines (blue for quail eggshells and orange for chiken eggshells) (**b**).

The X-ray diffraction patterns shown in Figure 8 indicate the presence, in all samples, of a compound with a high degree of crystallinity. This was identified as CaCO_3_ in a single phase, the characteristic Miller indices of the diffraction peaks being assigned using PDF file 01-086-4274.

By analyzing the diffraction maxima of plane (104) (Figure 8b), it is observed that the sample obtained from quail eggshells (denoted as QE-CS) tends to be located around the value of 29.45°. In contrast, the diffraction maxima for samples obtained from chicken eggshells shift to higher values (29.51°), indicating a short decrease in crystallite size.

Table 3 shows the values calculated using the Scherrer equation for each sample [58].

As can be seen from the low value of the intensity of the main maximum, the CE-CS sample presents a lower degree of crystallinity compared to the other analyzed samples, with the calculated crystallite size having a value of 52.21 nm. The sample obtained by grinding quail eggshells crystallizes predominantly in the direction given by plane (104), causing a slight increase in crystallite size up to 52.59 nm [58]. The adsorption of Zn^2+^ and Pb^2+^ ions on powders causes a slight increase in crystallite size but also the removal of non-crystalline residues, a fact indicated by the diminution of the XRD halo located around the values of 2θ = 10°.

**Figure 9:**
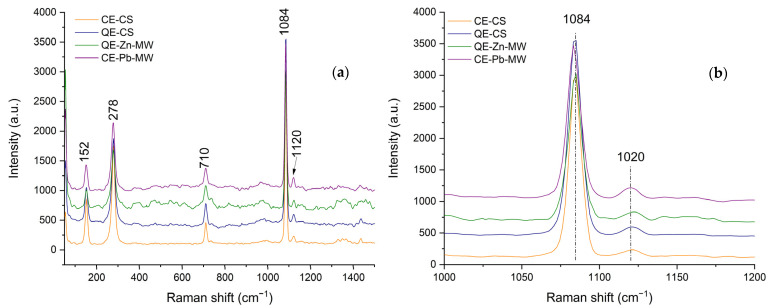
RAMAN spectra of chicken and quail eggshell powder (**a**) and CaCO_3_ (1084) characteristic band shift representation (**b**).

The RAMAN spectra obtained at room temperature, both for the samples obtained from chicken and quail eggshells (CE-CS and QE-CS) and for the samples on which Zn^2+^ and Pb^2+^ ions were adsorbed, are presented in Figure 9.

In agreement with the phases identified by X-ray diffraction, the most prominent bands are those specific to calcite (CaCO_3_). The most intense peak of the calcite phase (Figure 9b) appears at 1084 cm^−1^ and can be attributed to the symmetric stretching vibration ν1 [59,60]. The other intense peaks appear at 710 and 278 cm^−1^ and are due to ν4 (in-plane bending) of the CO_3_^2−^ group [60,61,62]. As the adsorption of Zn^2+^ and Pb^2+^ ions occurs, the characteristic bands shift to a smaller number of wavelengths, which may indicate an increase in structural disorder.

**Figure 10:**
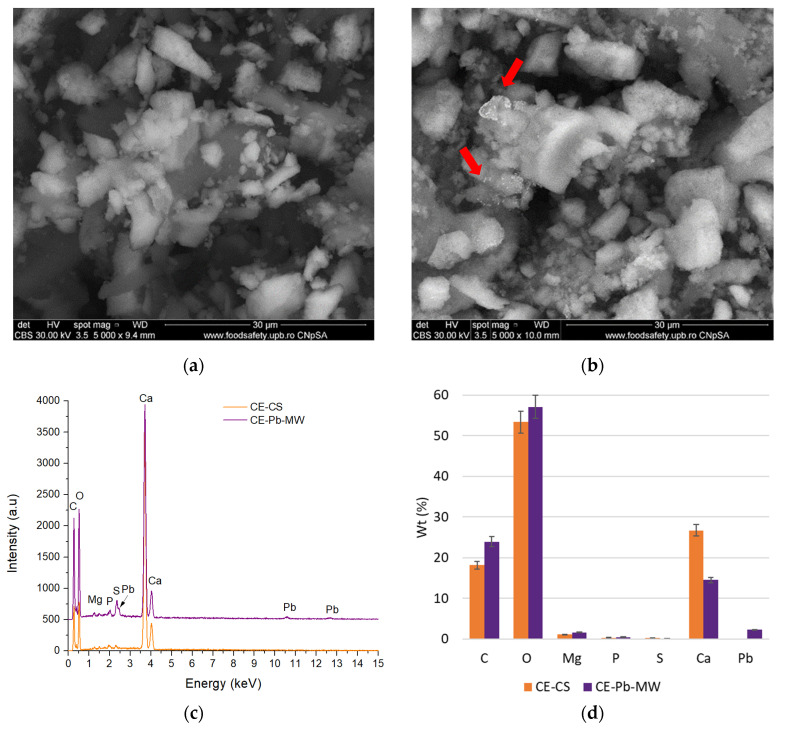
SEM micrographs of quail eggshell samples before Pb^2+^ biosorption (**a**) and after the biosorption process using MW (170 W) (**b**), with the corresponding EDS spectra before and after the biosorption process (**c**) and the percentage atomic composition of the selected microareas (**d**).

**Figure 11:**
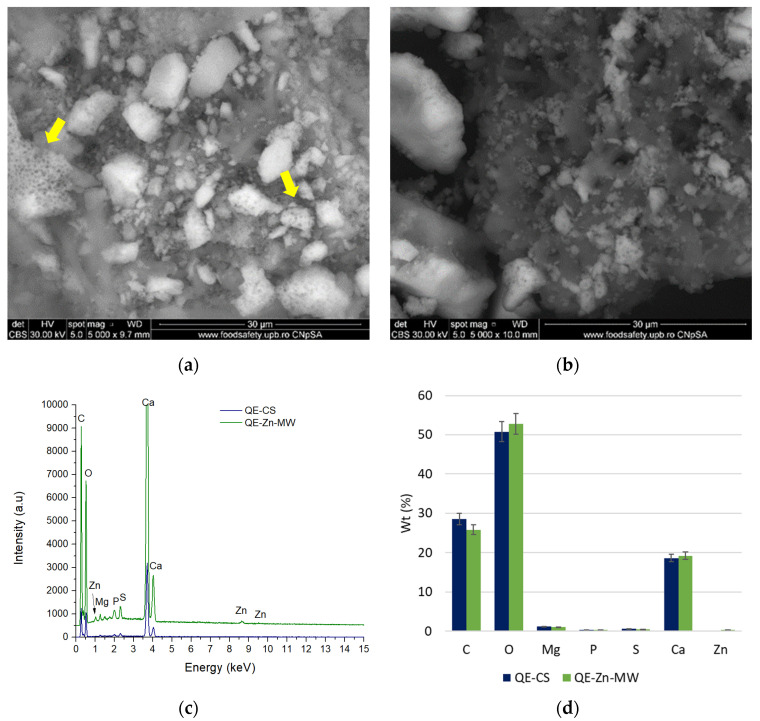
SEM micrographs of quail eggshell samples before Zn^2+^ biosorption (**a**) and after the biosorption process using MW (170 W) (**b**), with the corresponding EDS spectra before and after the biosorption process (**c**) and the percentage atomic composition of the selected microareas (**d**).

The morphology of the samples, both before impregnation and after contact with the ion solutions, was evaluated by SEM. Figure 10 presents the morphological aspects of the samples obtained from chicken eggshells, while the analyses performed on the samples obtained from quail eggshells are presented in Figure 11.

In the case of chicken eggshell samples, the particles are micron-sized with varied shapes and sharp corners (Figure 10a). As can be seen, the surface of these particles is irregular, which may contribute to the increase in the degree of metal adsorption on the surface [38,63,64]. This is confirmed by the image obtained after Pb adsorption (Figure 10b) where white areas can be highlighted that can be attributed to the element with higher Z (see red arrows).

The presence of Pb is also confirmed by the EDS spectra presented in Figure 10c, where the characteristic maxima of this element are observed compared to the samples analyzed before the contact of the powder with the solution containing Pb^2+^. Performing the compositional analysis on the selected microzones, a percentage of 2.24% Pb was identified that is deposited on the porous surface of the chicken eggshell particles (Figure 10d). The rest of the identified elements C, O, and Ca are characteristic of the chemical composition of the eggshell (CaCO_3_—previously identified by XRD).

The samples obtained from quail eggshells show a morphology similar to those from chicken eggshells, but in this case, the particles are more round and smaller [65], with some of the particles showing a pronounced porosity (Figure 11a—yellow arrows). The presence of the element adsorbed on the surface (Zn) is highlighted with the help of EDS spectra (Figure 11c). From a compositional point of view, these do not differ from chicken eggshells, being composed mainly of elements such as C, O, Mg, P, S, and Ca (Figure 11d).

There was almost no change in the surface morphology of chicken eggshells and quail powders before and after Pb/Zn adsorption, and only a few fine particles were observed on the surface of CE-Pb-MW and QE-Zn-MW samples.

## 4. Conclusions

In this study, we systematically analyzed the influence of different parameters for the Pb or Zn ions bioadsorption on untreated chicken, quail eggshells, and their mixture using a combination of various electrochemical and spectro-analytical techniques: pH-metry, conductometry/TDS, AAS, FTIR, RAMAN, XRD, and SEM-EDAX.

Under the investigated conditions, all types of unmodified eggshells proved a good capacity for the removal of Pb^2+^ and Zn^2+^ from aqueous solutions. The CEs were able to have more than 65% removal efficiency for lead and over 80% in the case of zinc for any type of agitation and temperature. Concerning the Zn removal efficiency of QEs, notable results were recorded when microwaves were applied (>90%) and at 40 °C for orbital shaking and ultrasound (>80%).

The present research revealed that the appropriate choice of the agitation mode allows for improving the adsorption process by increasing the removal efficiency. Taking into account the comparable removal efficiencies for different agitation techniques and the fact that the stirring time is reduced from 60 min (in the case of classical stirring) to 30 min (orbital stirring and ultrasound-assisted activation), respectively, and to 3 min (in the case of microwave-assisted activation), it can be stated that these activation/stirring techniques can be used successfully, especially in the case of zinc for all types of biosorbent. Thus, due to the time and energy savings, microwave-assisted activation represents a fast and efficient tool in the bioadsorption process of studied heavy metals using eggshells.

This study highlights the advantages of the applied agitation/activation systems, as well as the use of different unmodified eggshells (separately or in the mixture) as an absorbent: (i) simple method of preparation and use of the bioabsorbent; (ii) ecological; (iii) mild experimental conditions (r.t., pH 5); (iv) reduced working time and energy consumption; (v) cheap and efficient method for the valorization of food industry waste within the circular economy model for the treatment of wastewater containing heavy metals.

The overall results of the present study may offer new and valuable information for the optimal removal of Pb^2+^ and Zn^2+^ using unmodified eggshells, thus contributing to the sustainable management of waste through the recycling of this type of biomaterial.

## Figures and Tables

**Table 1:** The agitation technique conditions for the bioadsorption process.

Agitation/Activation Technique	Time [min]	Temperature	Other Parameters	Equipment Used
Classical agitation (CA)	60	r.t.	stirring speed 150 rpm	Magnetic laboratory stirrer—Nahita Blue, model 692, AuxiLab, Navarra, Spain
		40 °C		
Orbital agitation (OA)	30	r.t.	rotation speed 150 rpm	Stuart Orbital Incubator Platform Shaker Lab, SI50, Keison, Chelmsford, UK
		40 °C		
Ultrasound-assisted activation (US)	30	r.t.	-	Digital Pro Ultrasonic model PS-10A, Meditry Instrument Co., Ltd., Jiangsu, China
		40 °C		
Microwaves assisted activation (MW)	3	-	170 W	Microwave ovenn LG, MS2042DW, LG Electronics, Seoul, Republic of Korea
			340 W	

r.t.—room temperature.

**Table 2:** Physico-chemical parameters of initial solutions of Pb^2+^ and Zn^2+^.

Parameters	Pb^2+^	Zn^2+^
pH	5.52 ± 0.07	5.56 ± 0.03
EC [µS·cm^−1^]	10.46 ± 1.12	18.09 ± 2.61
TDS [ppm]	5.67 ± 1.10	8.80 ± 1.35
SAL [psu]	0.013 ± 0.001	0.010 ± 0.001

**Table 3:** XRD parameters for plane (104)’s characteristic of the CaCO_3_ crystalline phase.

Samples Name	2θ Position (°)	Intensity (a.u.)	d (Å)	FWHM (°)	Crystallite Size (nm)
CE-CS	29.51	2518	3.0265	0.1574	52.21 ± 0.01
QE-CS	29.45	6871	3.0317	0.1563	52.59 ± 0.01
QE-Zn-MW	29.51	6258	3.0265	0.1560	52.67 ± 0.01
CE-Pb-MW	29.45	5650	3.0316	0.1563	52.59 ± 0.01

## Data Availability

The original contributions presented in this study are included in the article. Further inquiries can be directed to the corresponding author.

## Supplementary Materials

The following supporting information can be downloaded at: https://www.mdpi.com/article/10.3390/ma18122794/s1, Table S1. Physico-chemical parameters determined for Pb^2+^ bioadsorption on eggshells. Table S2. Physico-chemical parameters determined for Zn^2+^ bioadsorption on eggshells. Table S3. Overall results obtained after Pb^2+^ and Zn^2+^ bioadsorption on eggshells. Table S4. Pearson correlations for removal efficiency taking into account each agitation mode. Table S5. Pearson correlation of removal efficiency for all samples. Figure S1. Influence of initial concentration of Pb^2+^ on the removal efficiency. Figure S2. Influence of contact time on the removal efficiency. Figure S3. Influence of initial pH on the removal efficiency. Figure S4. The point of zero charges of chicken eggshell powder.
