# Supplementary material for: The Influence of Different Parameters for the Removal of Pb and Zn Ions on Unmodified Waste Eggshells
Source: Materials (Basel). 2025 Jun 13;18(12):2794. doi: 10.3390/ma18122794 (PMC12195198; doi:10.3390/ma18122794)
Supplement: Supplementary file 1 [file materials-18-02794-s001.zip › materials-3647800-supplementary.pdf]

Supporting Information

The Influence of Different Parameters for the Removal of Pb and Zn Ions on Unmodified Waste Eggshells

Elena Petronela Bran [1], Oana-Irina Patriciu [2], Luminița Grosu [2], Irina-Claudia Alexa [2,*], Brîndușa Bălănucă [3], Adrian-Ionuț Nicoară [4,5] and Adriana-Luminița Fînaru [2,*]

[1] Doctoral School, "Vasile Alecsandri" University of Bacău, 157, Calea Mărăşeşti, 600115 Bacău, Romania
[2] Department of Chemical and Food Engineering, Faculty of Engineering, "Vasile Alecsandri" University of Bacău, 157, Calea Mărăşeşti, 600115 Bacău, Romania
[3] Department of Organic Chemistry "C. Nenitescu", Faculty of Chemical Engineering and Biotechnologies, National University of Science and Technology Politehnica Bucharest, 1–7, Gheorghe Polizu Street, 011061 Bucharest, Romania
[4] Department of Science and Engineering of Oxide Materials and Nanomaterials, Faculty of Chemical Engineering and Biotechnologies, National University of Science and Technology Politehnica Bucharest, 1–7, Gheorghe Polizu Street, 011061 Bucharest, Romania
[5] National Research Center for Micro and Nanomaterials, National University of Science and Technology Politehnica Bucharest, 060042 Bucharest, Romania
* Correspondence: irinaalexa@ub.ro (I.-C.A.); adrianaf@ub.ro (A.-L.F.); Tel.: +40-234-580170 (ext. 119) (I.-C.A.); +40-234-580170 (ext. 131) (A.-L.F.)

Table S1. Physico-chemical parameters determined for Pb^{2+} bioadsorption on eggshells.

Bioadsorbent type	Agitation technique	pH		EC [µS·cm^{-1}]		TDS [ppm]		SAL [psu]	
		r.t.	40 °C	r.t.	40 °C	r.t.	40 °C	r.t.	40 °C
Chicken eggshells	CA	7.88±0.05	7.87±0.06	98.87±2.21	99.67±1.98	48.78±0.56	48.88±0.42	0.049±0.003	0.051±0.002
	OA	7.81±0.04	7.58±0.03	73.41±1.53	82.15±1.14	35.99±0.48	40.33±0.49	0.040±0.002	0.044±0.002
	US	7.98±0.07	8.05±0.06	102.35±2.56	109.40±1.84	51.25±0.64	52.71±0.66	0.052±0.004	0.056±0.006
	MW	7.82±0.03	7.46±0.05	72.61±1.55	69.91±1.21	35.62±0.46	34.46±0.45	0.039±0.002	0.038±0.002
Quail eggshells	CA	7.93±0.12	7.89±0.11	100.14±2.55	99.24±2.21	49.53±0.59	48.92±0.57	0.050±0.005	0.053±0.005
	OA	7.81±0.09	7.64±0.05	73.61±1.62	87.38±1.91	36.21±0.48	42.79±0.51	0.041±0.003	0.046±0.003
	US	7.91±0.10	7.85±0.09	101.20±2.23	106.80±2.59	53.12±0.72	52.34±0.65	0.049±0.005	0.054±0.005
	MW	7.71±0.06	7.82±0.08	80.40±1.85	93.34±2.24	39.25±0.49	45.75±0.58	0.043±0.003	0.048±0.004
Mixture chicken and quail eggshells (ratio 1:1)	CA	7.92±0.10	7.87±0.09	99.20±2.08	99.14±2.21	48.54±0.55	48.97±0.62	0.041±0.003	0.051±0.006
	OA	7.80±0.08	7.57±0.07	73.72±1.55	77.52±1.76	35.98±0.48	38.03±0.54	0.039±0.003	0.041±0.004
	US	7.95±0.12	8.04±0.13	101.20±2.24	118.70±2.97	53.20±0.57	55.92±0.63	0.037±0.002	0.057±0.007
	MW	7.41±0.03	7.59±0.12	87.21±1.92	95.05±2.55	42.82±0.51	46.55±0.59	0.046±0.004	0.049±0.005

The data were expressed as mean ± standard deviation (SD).

Table S2. Physico-chemical parameters determined for Zn^{2+} bioadsorption on eggshells.

Bioadsorbent type	Agitation technique	pH		EC [μS·cm⁻¹]		TDS [ppm]		SAL [psu]	
		r.t.	40 °C	r.t.	40 °C	r.t.	40 °C	r.t.	40 °C
Chicken eggshells	CA	7.95±0.14	8.04±0.16	107.8±2.56	90.95±2.06	52.81±0.67	44.53±0.56	0.055±0.007	0.047±0.005
	OA	7.68±0.08	7.59±0.07	66.22±1.19	61.24±1.15	32.42±0.42	30.09±0.38	0.036±0.002	0.035±0.002
	US	8.10±0.18	8.01±0.15	68.99±1.20	100.70±2.42	33.81±0.44	49.41±0.65	0.038±0.002	0.052±0.006
	MW	7.55±0.06	7.65±0.10	105.20±2.55	78.68±1.75	52.69±0.58	38.55±0.47	0.045±0.004	0.042±0.003
Quail eggshells	CA	8.21±0.19	7.74±0.11	82.91±1.74	74.29±1.58	40.85±0.50	36.55±0.50	0.044±0.004	0.040±0.003
	OA	7.53±0.06	7.61±0.09	69.57±1.38	64.01±1.19	34.18±0.45	31.36±0.41	0.039±0.003	0.036±0.002
	US	8.07±0.18	7.93±0.13	85.08±1.85	70.08±1.32	41.73±0.48	34.33±0.46	0.045±0.004	0.038±0.002
	MW	7.71±0.09	7.76±0.12	80.40±1.86	80.80±1.89	39.25±0.48	42.21±0.50	0.043±0.003	0.043±0.004
Mixture chicken and quail eggshells (ratio 1:1)	CA	7.79±0.11	7.75±0.12	107.30±2.55	99.53±2.31	52.56±0.62	48.76±0.63	0.055±0.008	0.051±0.007
	OA	7.56±0.07	7.62±0.09	63.71±1.17	70.19±1.34	31.16±0.41	34.53±0.47	0.036±0.003	0.038±0.002
	US	8.01±0.13	7.90±0.12	75.09±1.68	125.00±3.27	36.79±0.49	61.25±0.84	0.040±0.004	0.063±0.009
	MW	7.65±0.08	7.60±0.08	77.24±1.76	99.08±2.45	37.90±0.51	48.45±0.62	0.041±0.004	0.047±0.005

The data were expressed as mean ± standard deviation (SD).

Table S3. Overall results obtained after Pb^{2+} and Zn^{2+} bioadsorption on eggshells.

Bioadsorbent type	Agitation / Activation technique	Codification sample	Removal efficiency R [%]	
			r.t.	40 °C
Chicken eggshells	CA	CE-Pb-CA	72.22±1.86	93.80±2.31
		CE-Zn-CA	92.70±2.31	97.56±2.44
	OA	CE-Pb-OA	85.36±2.02	65.40±1.57
		CE-Zn-OA	92.42±2.30	86.12±2.03
	US	CE-Pb-US	67.74±1.62	79.28±1.95
		CE-Zn-US	83.04±1.98	84.72±2.01
	MW	CE-Pb-MW	85.98±2.06	75.20±1.87
		CE-Zn-MW	81.36±1.97	83.84±1.99
Quail eggshells	CA	QE-Pb-CA	49.78±1.52	95.66±2.40
		QE-Zn-CA	98.20±2.48	98.02±2.43
	OA	QE-Pb-OA	75.70±1.90	81.94±1.97
		QE-Zn-OA	72.40±1.86	83.20±1.99
	US	QE-Pb-US	51.12±1.55	63.56±1.62
		QE-Zn-US	83.84±1.98	80.84±1.91
	MW	QE-Pb-MW	47.54±1.50	51.16±1.57
		QE-Zn-MW	90.10±2.24	90.80±2.26
Mixture chicken and quail eggshells (ratio 1:1)	CA	CE+QE-Pb-CA	63.54±1.56	93.88±21.33
		CE+QE-Zn-CA	97.68±2.46	98.04±2.41
	OA	CE+QE-Pb-OA	81.06±1.95	83.44±1.96
		CE+QE-Zn-OA	79.68±1.88	82.84±1.88
	US	CE+QE-Pb-US	51.18±1.56	56.36±1.62
		CE+QE-Zn-US	85.68±2.05	89.76±2.17
	MW	CE+QE-Pb-MW	78.12±1.93	62.72±1.60
		CE+QE-Zn-MW	74.70±1.89	89.26±2.15

Table S4. Pearson correlations for removal efficiency taking into account each agitation mode.

CA	Pb (r.t.)	Pb (40 °C)	Zn (r.t.)	Zn (40 °C)	OA	Pb (r.t.)	Pb (40 °C)	Zn (r.t.)	Zn (40 °C)
**Pb (r.t.)**	**1.00**				**Pb (r.t.)**	**1.00**			
**Pb (40 °C)**	-0.84	**1.00**			**Pb (40 °C)**	**0.98**	**1.00**		
**Zn (r.t.)**	-0.94	0.60	**1.00**		**Zn (r.t.)**	-0.79	-0.90	**1.00**	
**Zn (40 °C)**	-0.77	**0.99**	0.50	**1.00**	**Zn (40 °C)**	0.77	**0.89**	-1.00	**1.00**
**US**	**Pb (r.t.)**	**Pb (40 °C)**	**Zn (r.t.)**	**Zn (40 °C)**	**MW**	**Pb (r.t.)**	**Pb (40 °C)**	**Zn (r.t.)**	**Zn (40 °C)**
**Pb (r.t.)**	**1.00**				**Pb (r.t.)**	**1.00**			
**Pb (40 °C)**	-0.73	**1.00**			**Pb (40 °C)**	-0.90	**1.00**		
**Zn (r.t.)**	**0.95**	-0.91	**1.00**		**Zn (r.t.)**	**0.94**	-0.69	**1.00**	
**Zn (40 °C)**	-0.07	**0.73**	0.50	**1.00**	**Zn (40 °C)**	-0.80	0.45	-0.96	**1.00**

Table S5. Pearson correlation of removal efficiency for all samples.   .

	Pb (r.t.)	Pb (40 °C)	Zn (r.t.)	Zn (40 °C)
**Pb (r.t.)**	**1.00**			
**Pb (40 °C)**	-0.42	**1.00**		
**Zn (r.t.)**	0.22	0.30	**1.00**	

Preliminary Study for Establishing the Optimal Conditions for the Adsorption Process

The preliminary study was conducted on Pb^{2+} biosorption on chicken eggshell powder in order to establish the optimal parameters: initial concentration of the metal ion, biosorbent amount, solution pH and the contact time.

Therefore, the initial concentration of the metal ion (Pb^{2+}) was varied between 1.31 and 6.94 mg·L^{-1}.

It was found that the optimum of the bioadsorption process was reached at an initial concentration of the metal ion of 5 mg·L^{-1} (Figure S1).

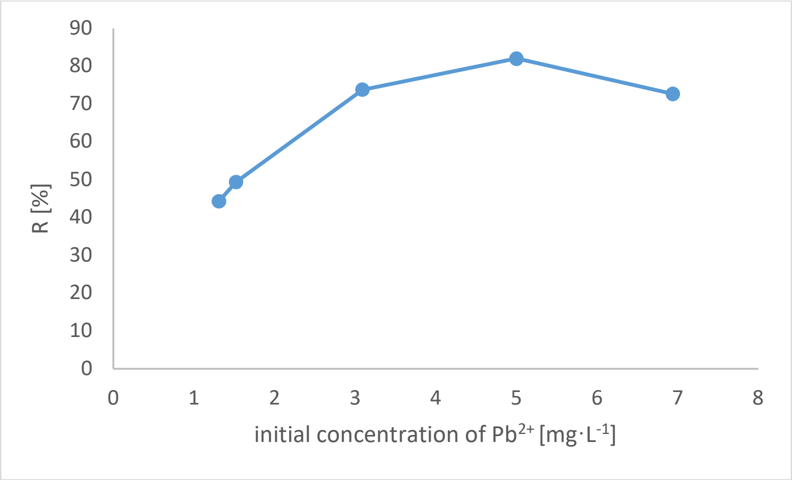

Figure S1. Influence of initial concentration of Pb^{2+} on the removal efficiency.

The contact time between the two phases, necessary to reach equilibrium, is another important parameter of the biosorption process. This is determined by the fact that a long contact time leads to an increase in the costs of the biosorption process, while short contact

time can lead to a drastic decrease in the efficiency of the biosorption process. The optimum value for the contact time for the classical agitation at room temperature was establish to 60 minutes (Figure S2).

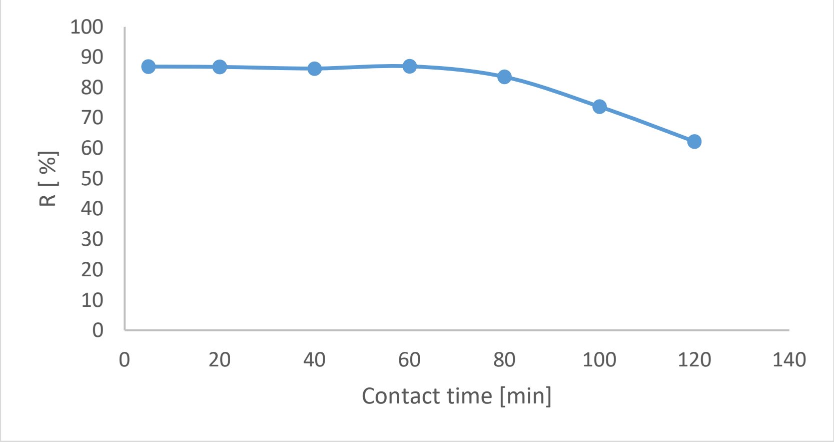

Figure S2. Influence of contact time on the removal efficiency.

The biosorbent characteristics of a material can be influenced by the initial pH of the aqueous solution of adsorbate. As a result of the preliminary study, in which the pH was varied between 3 and 7, the optimum pH value for a maximum bioadsorption capacity was established at 5 (Figure S3).

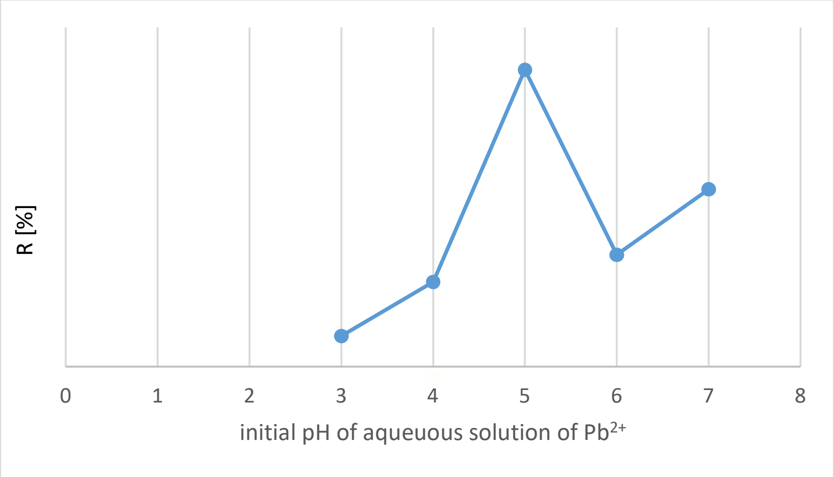

Figure S3. Influence of initial pH on the removal efficiency.

Previously, the determination of the point of zero charges of chicken eggshell powder was realized using the method described by Praipipat et al. [39]. The pH_{pzc} = 4.63, so $pH_{solution} > pH_{pzc}$.

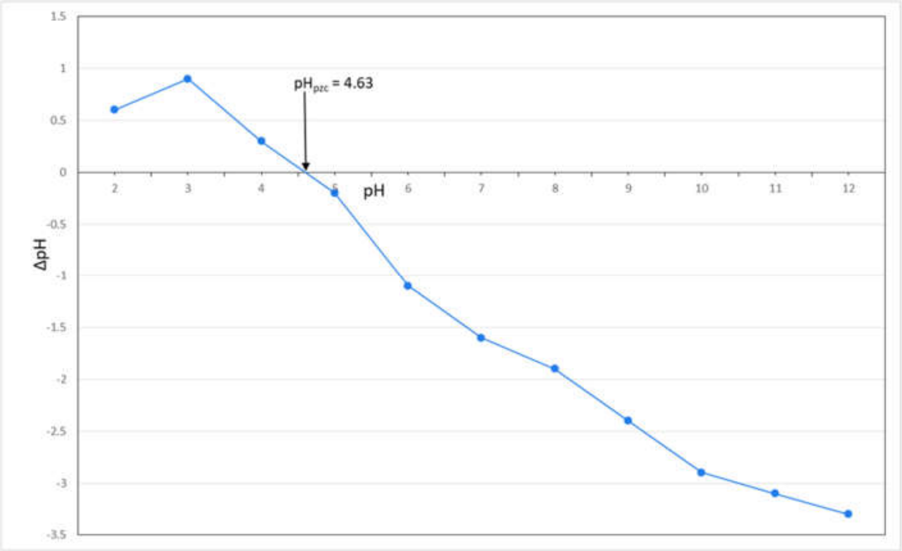

Figure S4. The point of zero charges of chicken eggshell powder.
